# Progressive Achilles Loading via Clinician e-Support (PACE): Protocol for a Dual-Site Randomized Controlled Trial

**DOI:** 10.2196/90955

**Published:** 2026-04-07

**Authors:** Micah A K M Wong, Mathew Frazier, Ryan Jensen, Timothy Fleagle, Barbara Van Gorp, Nicole Hall, Patrick Ten Eyck, Linder Wendt, Molly Pacha, Jessica Danielson, Mederic Hall, Jason Wilken, Ruth L Chimenti

**Affiliations:** 1 Department of Physical Therapy and Rehabilitation Science, Carver College of Medicine, University of Iowa, 500 Newton Rd., Iowa City, IA, United States, 1 3193359802; 2 Department of Rehabilitation and Physical Therapy, Carl R. Darnall Army Medical Center, Fort Hood, TX, United States; 3 Department of Orthopedics and Rehabilitation, University of Iowa, Iowa City, IA, United States; 4 Institute for Clinical and Translational Science, University of Iowa, Iowa City, IA, United States

**Keywords:** military medicine, tendinopathy, exercise therapy, telemedicine, delivery of health care

## Abstract

**Background:**

Achilles tendinopathy (AT) is a common condition that limits activity participation in both active and sedentary populations. While exercise and education are well-established treatments for AT, information is lacking on the amount of intervention required and the prognostic factors associated with response to noninvasive treatments.

**Objective:**

This noninferiority randomized controlled trial will determine the efficacy of a single-visit, physical therapist (PT)–initiated rehabilitation program compared to a multivisit, PT-guided telehealth rehabilitation program for AT and will identify early prognostic factors for individuals who experience the greatest improvement in pain and disability by 4 weeks.

**Methods:**

In this single-blind, 2-arm, parallel-group trial, 160 individuals with AT will be randomized to either a single-visit PT-initiated rehabilitation program or a multivisit, PT-guided telehealth rehabilitation program. Primary outcomes will be the numeric rating scale for movement pain and the Victorian Institute of Sport Assessment–Achilles score for disability. Secondary outcomes will include measures of tendon health, measures within the psychosocial health domain, and military readiness.

**Results:**

This research was funded in July 2024. Participant enrollment began in August 2025 and is expected to conclude in 2028. As of January 2026, 15 participants have been enrolled at the University of Iowa Hospitals and Clinics, meeting the recruitment goals.

**Conclusions:**

The PACE (Progressive Achilles Loading via Clinician e-Support) trial will identify a rehabilitation program focused on education and exercise for AT that expands access to care and will identify factors that predict responses to exercise and education.

## Introduction

Achilles tendinopathy (AT) can severely limit activity participation across the general [1], athletic [2], and military [3] populations. The overall prevalence of AT is between 4% and 7% in the general population [4], with a higher prevalence in middle-aged adults and athletes [5]. Exercise and education interventions have been shown to be effective in decreasing pain and improving function [6] across active and sedentary populations [4] with AT.

While care for AT typically involves an average of 4 visits (range 1-31) with a physical therapist (PT) [7], the evolving civilian health care landscape, marked by an aging population, rising costs, and rural access challenges, demands innovative approaches to care delivery [8,9]. Military health care faces similar barriers to care in high–operational tempo garrison, training, and deployed environments. Large-scale combat operations against near-peer adversaries will necessitate prolonged casualty care, of which telehealth is a component. Telehealth has emerged in the post–COVID-19 pandemic era as a valid [10,11] and accessible [12] touchpoint for health care access. The US Military Health System made “virtual first” the goal of its restructuring plans, following the trends of many health systems [13]. Telehealth noninferiority has been previously demonstrated for AT [6], total knee replacement rehabilitation [14,15], and general musculoskeletal care [16]. Creating a treatment protocol revolving around the independent management of AT using evidence-based exercise and education interventions, while using telehealth guidance by health care professionals, will expand access to high-quality rehabilitative care.

Recent evidence has shown that overtreatment of musculoskeletal disorders does not lead to improved outcomes. Hopewell et al [17] found that a single session of education and self-management advice with a PT was noninferior to 6 sessions of in-person progressive exercise in improving pain and function among individuals with subacromial shoulder pain. Similarly, Katz et al [18] found that a self-directed home exercise plan alone was noninferior to 14 sessions of PT-guided exercise and manual therapy for pain reduction in individuals with meniscal tear–related knee pain. AT is lacking in trials to gauge the minimal effective dosage of exercise interventions. Pivoting to expand access through novel care delivery pathways is now more feasible, given the evidence that pain and function can improve with focused minimal care [19].

Clinicians currently lack evidence-based prognostic factors to differentiate patients who will benefit from rehabilitation from those who are less likely to respond [20,21]. Given that 26% to 47% of individuals with AT proceed to injections or surgery [22-24], the identification of prognostic factors could inform more efficient and personalized care. For nonresponders to rehabilitation, delays in progressing to invasive treatments prolong Achilles tendon pain, waste patient time and health care resources, and increase the total number of limited duty days as multiple treatments are tried. There is a critical need for a large, prospective study to identify factors that predict which patients with AT are most likely to respond to rehabilitation.

This trial’s objective is to identify a rehabilitation program focused on education and exercise for AT that expands access to care and to identify factors that predict responses to rehabilitation. Our aims are twofold: (1) to determine the efficacy of a single-visit, PT-initiated rehabilitation program compared to a multivisit, PT-guided telehealth rehabilitation program for AT and (2) to identify early prognostic factors for individuals who experience the greatest improvement in pain and disability by 4 weeks. We hypothesize that a single-visit, PT-initiated rehabilitation program with a self-guided intervention will be as effective in improving pain and disability as a multivisit, PT-guided telehealth intervention. We also hypothesize that individuals with greater immediate (within 2 weeks) improvements in self-efficacy, pain, and disability will obtain the greatest benefits from rehabilitation.

## Methods

### Overview

Progressive Achilles Loading via Clinician e-Support (PACE) is a single-blind, noninferiority, 2-arm, parallel-group randomized controlled trial. Individuals with AT will be randomized to a single-visit, PT-initiated rehabilitation program or to a multivisit, PT-guided telehealth rehabilitation program. All participants will initiate care with a single synchronous in-person visit with a PT and have access to information on exercise and education materials via an online learning management system. Primary outcomes will be the numeric rating scale (NRS) for movement pain and the Victorian Institute of Sport Assessment–Achilles (VISA-A) score for disability.

We will enroll 160 individuals with AT across 2 sites: 1 military treatment facility and 1 civilian academic center. Outcomes will be assessed at 2, 4, 8, 12, 26, and 52 weeks, with the primary end point at week 4. Deviations to the protocol will be reported in real time on the Open Science Framework. This protocol is reported in accordance with the SPIRIT (Standard Protocol Items: Recommendations for Interventional Trials) guidelines [25].

### Participant Selection

Participants will be recruited and enrolled from 2 sites: the Carl R. Darnall Army Medical Center and the University of Iowa Hospitals & Clinics (UIHC). Active and reserve military personnel, veterans, and civilians will be eligible with recruitment carried out at site-specific clinics (family medicine, physical therapy, and sports medicine); on social media; through email requests; and through the distribution of brochures and flyers in community areas. A full list of the inclusion and exclusion criteria is provided in Textbox 1. To allow for the consideration of sex as a biological variable in the study findings, we will target at least 33% enrollment of female participants across both sites, with an anticipated higher recruitment rate of female participants at the UIHC to compensate for an anticipated lower rate of female recruitment at the CRDAMC (Carl R Darnall Army Medical Center, Fort Hood).

Textbox 1. **Eligibility criteria for study enrollment across both sites: the Carl R. Darnall Army Medical Center and the University of Iowa Hospitals and Clinics.**

**Inclusion criteria**

- Pain localized to the Achilles tendon (insertional or midportion)
- Achilles pain ≥3 out of 10 with a tendon-loading exercise

**Exclusion criteria**

- Age <18 y or >60 y
- BMI >45 kg/m^2^
- Pain primarily due to a differential diagnosis, including paratendonitis, bursitis (retrocalcaneal or subcutaneous), posterior ankle impingement or os trigonum, irritation or neuropathy of the sural nerve, or plantaris tendon involvement
- Presence of a partial Achilles tendon tear or rupture on imaging, or a history of Achilles tendon rupture that was verified by surgical or conservative management
- Attendance at physical therapy for Achilles tendinopathy (AT) within the past 3 mo
- History of a steroid injection to lower-extremity tendons or fascia, extracorporeal shock wave therapy, or any injection to the Achilles tendon region within the past 3 mo
- History of fluoroquinolone use within the past 6 mo
- History of surgery or an invasive procedure for AT on the side enrolling for treatment
- Diagnosis of systemic conditions affecting the foot and ankle (eg, rheumatoid arthritis and ankylosing spondylitis), an endocrine disorder with complications (eg, uncontrolled type 1 or type 2 diabetes and diabetic peripheral neuropathy), a connective tissue disorder (eg, Marfan syndrome and Ehlers-Danlos syndrome), peripheral vascular disease, or pregnancy
- High risk for falls (four square step test >15 s)
- Refusal of randomization or inability or uninterestedness in completing virtual visits using a webcam or smartphone, or in completing quizzes and surveys

This study will be stopped prior to its completion if recruitment challenges significantly affect the ability to complete analyses of the primary outcomes. Therefore, we anticipate that it is more likely that we need to add and modify recruitment strategies rather than invoke this stopping rule.

### Screening

We will follow a 3-stage screening process at the CRDAMC. First, a member of the study team will review new referrals to CRDAMC physical therapy for lower leg, ankle, and foot conditions. Second, study staff will review the past medical history of potentially eligible participants as part of the referral review process. If eligibility criteria are potentially met, a PT will contact the patient by phone to arrange a Health Insurance Portability and Accountability Act (HIPAA)–compliant video meeting to review the eligibility criteria in more detail and have the participant perform tendon-loading exercises to determine movement-evoked pain intensity localized to the Achilles tendon. The final stage of the screening process includes an in-person clinical examination to confirm the diagnosis of AT and a review of the eligibility criteria.

At the UIHC, we will use a similar 3-stage screening process. First, all recruited potential participants will complete a brief online eligibility survey. Second, if the criteria are potentially met, a PT will meet with them over a video meeting to review the eligibility criteria in more detail and have them perform tendon-loading exercises to determine movement pain intensity localized to the Achilles tendon. The final stage of the screening process includes an in-person clinical examination to confirm the diagnosis of AT and a review of the eligibility criteria.

### Randomization and Concealed Allocation

Individuals who meet all eligibility criteria during the in-person visit will be randomized to either a single-visit or multivisit rehabilitation intervention. Randomization will occur at a 1:1 ratio with stratification at each site for each treatment arm using permuted random blocks. Stratification variables will be based on AT type (insertional or midportion) and symptom duration (acute <3 months or chronic >3 months), which will result in 4 strata per site. Variable block sizes of 2, 4, and 6 will be used so that randomization blocks cannot be guessed. To minimize selection bias, group assignments will only be revealed after eligibility is confirmed, all baseline measures have been collected, and the in-person treatment visit has been completed. The PTs enrolling participants will not have access to the allocation sequence, which will be concealed using a computer-based randomization system in REDCap (Research Electronic Data Capture). Unblinding of group allocation will occur in the case of serious adverse events or unanticipated problems related to the intervention, as decided by the safety officer (MH) in conjunction with the steering committee (RLC, JW, and MF).

### Exercise and Education Intervention

**Figure 1.**
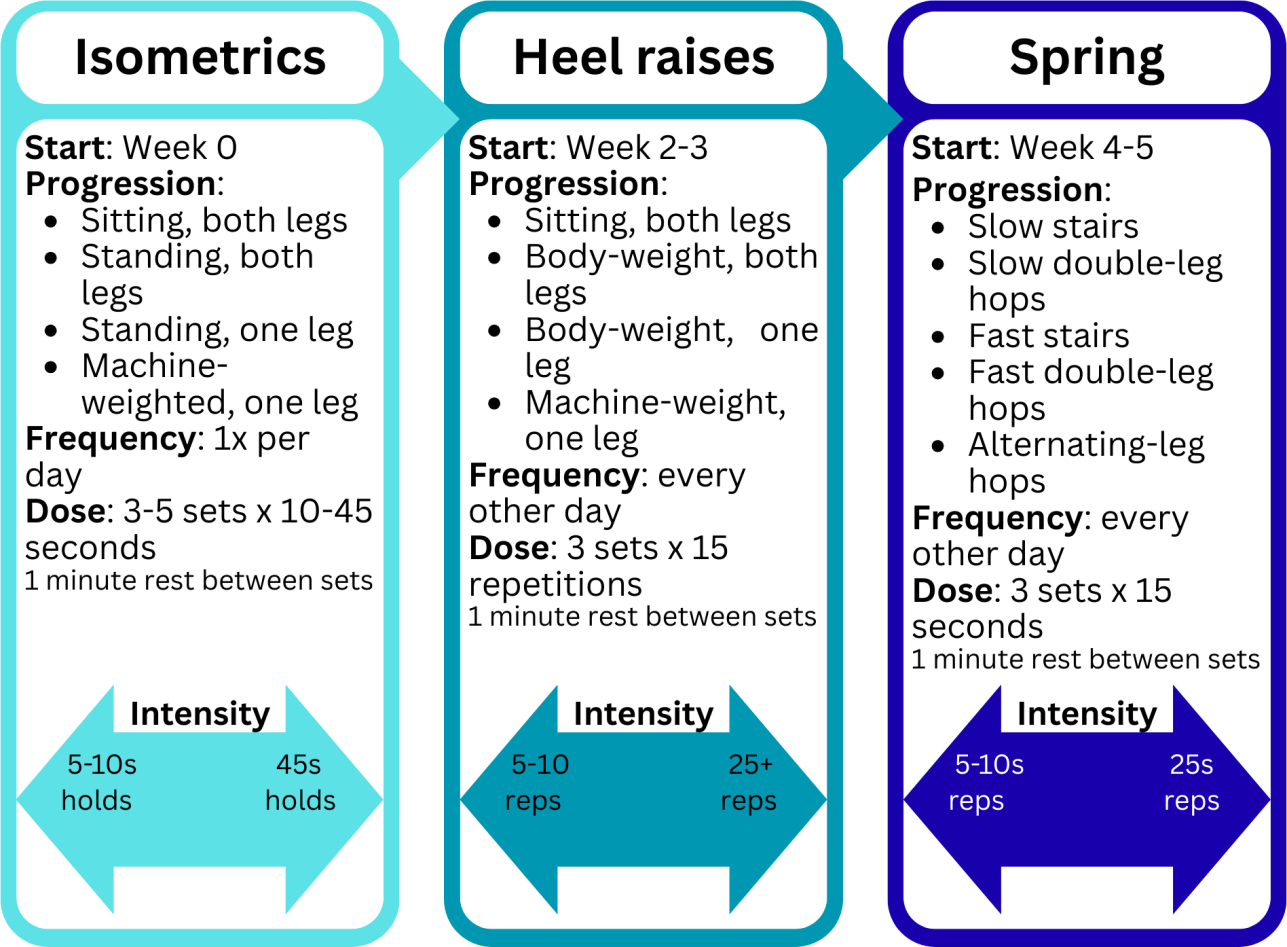
Exercise progression performed in both the single-visit and multivisit trial arms. Exercises are divided into 3 phases—isometric, heel raise, and spring—with time points, progression, frequency, dose, and intensity modifications displayed.

Both arms of this trial will be using an online learning management system (TalentLMS; Epignosis) for education on the pathophysiology, self-management, and exercise progression for AT. This exercise and education content has been shown to be equally effective in reducing pain when delivered by telehealth compared to in-person treatment for AT [26,27]. For this study, the learning units (Table 1) are either completed independently at a self-paced rate (single-visit group) or reviewed during weekly sessions with a PT (multivisit group). The learning units include information about the home exercise program and provide guidance on progression. The exercise component has been previously described by Post et al [27] and is shown in Figure 1.

**Table 1.** Education and exercise content organized into 6 successive learning units to address common questions about Achilles tendinopathy.

Unit	Unit name	Questions addressed
1	From diagnosis to recovery	What is Achilles tendinopathy? How is Achilles tendinopathy diagnosed? Is stiffness normal in Achilles tendinopathy? Can exercise reduce Achilles tendinopathy pain and stiffness? What’s the best type of exercise to start with for Achilles tendinopathy?
2	Factors linked with Achilles tendinopathy	Why is my tendon so swollen? Would a bone spur or calcification affect my physical therapy? Does a Haglund’s deformity cause Achilles tendinopathy pain? Should I get imaging of my Achilles tendon? Am I ready to progress to Phase 2 of the home exercise program?
3	Achilles tendinopathy pain: factors beyond the tendon	What causes Achilles tendon pain? How does mental load change your Achilles tendon pain? How can you use pain as a guide to recovery for Achilles tendinopathy?
4	Progressive loading exercises for Achilles tendinopathy	How does tendon loading in daily life differ from this exercise program? How can you use load to increase tendon capacity? What would happen if you don’t treat or do exercise for Achilles tendinopathy? How do I know when I’m ready to progress to Phase 3 of the home exercise program?
5	Benefits of physical activity	How does physical activity help your body recover? What is the recommended amount of exercise? How do you stay active with Achilles tendinopathy pain?
6	Recognizing recovery	How long does recovery from Achilles tendinopathy take? What are common signs of recovery? What are other treatment options? When is surgery recommended for Achilles tendinopathy?

### Single-Visit Group

Participants in the single-visit, PT-initiated group will undergo a self-paced rehabilitation program initiated with 1 synchronous, in-person visit with a PT. At the first visit, participants will be instructed to complete the 6 successive online learning units over an 8-week period, with flexibility to choose the timing that works best for them.

### Multivisit Group

Participants in the multivisit, PT-guided rehabilitation program will have 7 one-on-one telehealth visits over 8 weeks with a PT. These 30-minute visits will include exercise participation and education. Participants will be expected to complete 1 educational unit per week and will be given an exercise log to track exercises completed at home between treatment sessions. Instruction on exercise performance and progression will be provided by a PT throughout the plan of care. The PT will review the weekly educational content with the participant at each visit.

### Outcome Measures

To maintain blinding of outcome assessment, baseline measures will be gathered in person prior to randomization. A research assistant blinded to the treatment groups will capture outcomes after randomization. Participants will complete 3 survey-only evaluation sessions at 2, 12, and 26 weeks. Participants will complete 3 video evaluation sessions, including surveys, at 4 weeks (primary end point), 8 weeks, and 52 weeks. The video evaluation sessions will be used to capture pain with tendon-loading activities (heel raises and hops) in real time, assess heel raise performance (number of heel raises), and screen for adverse events. A visualization of measure administration can be seen in Table 2.

**Table 2.** Data collection schedule and format for both the self-guided and clinician-guided intervention groups over the duration of study participation.

Format of data collection	In person	Remote					
	Before the intervention	During the intervention			After the intervention		
	Week 0	Week 2	Week 4^a^	Week 8	Week 12	Week 26	Week 52
Randomization	✓						
Tendon Health Domains (Performance-based)	✓		✓	✓			✓
Tendon Health Domains (Survey-based)	✓	✓	✓	✓	✓	✓	✓
Ultrasound imaging	✓						
Clinical variables	✓		✓	✓	✓	✓	✓

a Week 4 indicates primary end point.

### Primary Outcomes

The primary outcomes of this study are movement pain and disability (Table 3). Movement pain will be measured using the NRS. Assessing the participant’s worst pain during single-limb heel raises allows for comparison of outcomes with previous clinical trials for AT [26,28-30]. Disability will be measured using the VISA-A, which assesses disability specific to AT and allows comparison to other studies, as this is the most commonly used self-reported outcome for AT [31-34].

**Table 3.** Measures and psychometric properties used in the PACE (progressive Achilles loading via clinician e-support) study^a^.

Health domain	Measure	Psychometric properties
Pain	Pain with movement during single-limb heel raises [26,28-30] and hops^b^^,^^c^ Pain at rest^c^	MID^d^ 1.1 (95% CI 0.9-1.6) [35] on a 0‐10 NRS^e^ MID for PACE1.7 [36] on a 0‐10 NRS
Disability	VISA-A^b^^,^^f^^,^^g^ [37,38] Tendinopathy severity assessment^c^ [39]	MID 8.2 (SD 16.3) [40] Cronbach *α*=0.808 Reliability: ICC^h^ 0.930 (95% CI 0.881-0.959) Standard error of measurement=6.54 units [41]
Physical function and capacity	PROMIS^f^^,^^i^ physical function CAT^j^ [42] Maximum number of single-limb heel raises^c^ [43] Heel raise work^c^	Internal consistency: person reliability=0.96 Item reliability=0.99 [44] Test-retest reliability: ICC 0.83 (95% CI 0.63-0.92) [45] Interrater reliability: ICC 0.99 (95% CI 0.88-0.99) Criterion validity: ICC 0.97 [46]
Psychosocial factors	Tampa Scale of Kinesiophobia (17-item)^f^ PROMIS Self-Efficacy CAT^f^ [42] Optimal Screening for Prediction of Referral and Outcome-Yellow Flag tool (17-item)^f^ PROMIS Sleep Disturbance CAT^f^ [47]	Cronbach *α*=0.70‐0.83 Test-retest reliability: *r*=0.64‐0.80 Concurrent validity: *r*=0.33‐0.59 [48] Concurrent validity with legacy measures: *r*=0.59‐0.76 [49] Test-retest reliability: ICC 0.88; *P*<.001 [50] Internal consistency, marginal reliabilities=0.92 Test-retest reliability, *r*=0.80 [47]
Patient rating of condition	Patient Acceptable State^f^ [51] Patient Global Rating of Change^f^ [52]	Concurrent validity [53] PROMIS Pain Interference <56.0 (AUC^k^ 0.940) PROMIS Pain Intensity <48.4 (AUC 0.936) PROMIS Physical Function >44.7 (AUC 0.883) Test-retest reliability: ICC 0.90 Face validity: Pearson *r*=0.72‐0.90
Participation in life activities	PROMIS Ability to Participate in Social Activities CAT^f^ [54] PROMIS Pain Interference CAT^f^ [44]	Reliability >0.90 Sufficient unidimensionality root mean square error approximation=0.108 Cronbach *α*=0.96 [55] Concurrent validity with brief pain inventory: *r*=0.76 (95% CI 0.64-0.85); *P*<.01
Quality of life	World Health Quality of Life–2^f^ [56] (subset of the first 2 items on the WHOQOL-BREF^l^)	Cronbach *α*=0.51-0.82 for entire WHOQOL-BREF

a Primary outcomes are movement pain as measured with the NRS and disability measured with the VISA-A.

b Primary outcome.

c Tendon health domain (performance-based measure).

d MCID: minimal important difference.

e NRS: numeric rating scale.

f Tendon health domain (survey-based measure).

g VISA-A: Victorian Institute of Sport Assessment–Achilles.

h ICC: intraclass correlation coefficient.

i PROMIS: patient-reported outcome measure information system.

j CAT: computer adaptive test.

k AUC: area under the curve.

l WHOQOL-BREF: World Health Organization Quality of Life–Brief.

### Secondary Outcomes

At baseline, we will capture demographics, military status, and tendon anterior-posterior diameter using ultrasound imaging [57]. In alignment with the core domain of tendon health, as defined by the International Scientific Tendinopathy Symposium Consensus [58], we will capture additional measures of pain and disability, along with measures of participation in life activities, physical function and capacity, psychological factors, patient rating of the condition, and quality of life over time (Table 3). Activity level will be captured using the Tegner scale [59], minutes per week spent doing moderate and vigorous physical activity, and questions about military readiness (eg, ability to complete a 2-mile run, wear military boots, and wear load-bearing equipment). Potential confounders that may influence the response to rehabilitation will also be captured, including the use of cointerventions or medications, self-reported exercise logs, and completion of educational units as captured in the learning management system. To assess treatment credibility, participants will complete a series of items evaluating the intervention’s impact on their perspective, understanding of self-management strategies, clarity of information, and appropriateness of the approach for managing AT, rated on a 5-point Likert scale (0=not at all, 1=a little bit, 2=somewhat, 3=quite a bit, and 4=very much).

### Qualitative Measures

To inform the feasibility of translating these rehabilitation strategies to a battlefield setting, we will use purposive sampling to invite participants at the CRDAMC who rotate to the field to participate in focus groups to discuss barriers and facilitators to participation in the rehabilitation programs. Participants will be invited to participate in the qualitative interviews until data saturation is reached. Semistructured interviews will be held via a videoconference platform that allows for HIPAA-compliant audio recording of the interviews. All audio recordings will be transcribed verbatim.

### Safety Monitoring and Reporting

Exercise and education interventions are considered minimal risk by the University of Iowa Institutional Review Board (Biomedical IRB-01) and do not exceed the risk associated with standard clinical care; therefore, this study uses a safety officer (MH) rather than a data and safety monitoring board. The data and safety of participants will be monitored regularly by the intervention clinicians and outcome assessors, who will be contacting both the self-paced and PT-guided participants throughout the duration of study participation. All adverse events, serious adverse events, and unanticipated problems will be documented in REDCap by study team members and reviewed regularly by the safety officer. Any potential referrals for appropriate care for participants who report considerable pain or discomfort will be discussed with the safety officer and followed up in a timely manner.

### Power Calculation

The trial is powered to evaluate noninferiority between the two treatment groups at 4 weeks for 2 coprimary outcomes: (1) movement pain on a 0‐10 NRS, using a 1.1-point noninferiority margin, and (2) disability using the VISA-A, with an 8.2-point noninferiority margin. Both margins are consistent with previously published clinically meaningful thresholds [40]. Because the primary analysis will evaluate both outcomes using separate noninferiority tests, we applied a Bonferroni-adjusted, 2-sided *α*=.025 for each outcome. Power calculations assume comparison of mean group differences at 4 weeks using the same linear mixed-effects models planned for the primary analysis with adjustment for baseline values. A sample of 64 participants per group provides ≥80% power to conclude noninferiority for each outcome at *α*=.025. To account for an anticipated 20% dropout, we inflated the sample size to 80 participants per group.

### Data Analysis

Baseline characteristics will be presented as counts and percentages for categorical measures. Continuous measures will be reported as means and SDs or medians and IQRs, as appropriate. Summary statistics by group will be provided for the primary and secondary outcomes and will be compared using 2-sample *t* tests, Wilcoxon rank-sum tests, or Fisher exact tests, as appropriate. Our intention-to-treat analysis will use linear mixed-effects models to assess the noninferiority of the single-visit intervention compared to the multivisit intervention, adjusting for baseline values of the primary outcomes. Additional intention-to-treat analyses will include any covariates that differed between groups at baseline. Primary outcomes will be the 4-week changes in measures of pain and function, and models will account for site using a random effect. Point estimates, CIs, and *P* values will be obtained for between-group differences in 4-week mean change compared to the margin of noninferiority. Significance for testing pain and function will be assessed at an *α* level of .025. We plan to report all data disaggregated by sex, AT type, and AT duration.

Secondary analyses will use linear mixed-effects models to examine predictors of improvement in pain and disability separately at 4 weeks. The same analysis will be performed at 12, 26, and 52 weeks as secondary end points. Outcomes will be the changes measured at each follow-up time point, and the models will be controlled for the corresponding baseline values. Model selection will be conducted to identify the optimal predictor set for each change outcome over time. The pool of candidate predictors includes baseline demographic and ultrasound imaging variables, as well as immediate improvement (within 2 weeks) of pain-related psychological factors, knowledge on self-management, pain, and disability. Models for all additional predictor combinations up to order 3 will be fit, and the Akaike information criterion will be calculated to compare model fits with the same outcome. Point estimates, CIs, and *P* values will be reported for predictors in models selected by the Akaike information criterion for each outcome. Significance for model predictors will be assessed at an *α* level of .05. Categorization of continuous variables using clinically meaningful cut points, such as obesity categories for the continuous BMI variable, will also be explored.

### Missing Data

Reasons for missing data will be recorded and compared qualitatively between participants who drop out and participants who complete the study. Participant characteristics collected prior to dropout will be compared to those of participants who complete the study. For the primary imputation strategy, if comparisons show that participant characteristics do not differ meaningfully between those with and without missing data, we will assume the data are missing at random and proceed accordingly. Under this assumption, we will use multiple imputation of the missing values for variables included in the linear mixed-effects models. Multiple imputation will be performed using SAS PROC MI and PROC MIANALYZE procedures in SAS software (version 9.4; SAS Institute Inc).

All variables in the primary analyses and, if feasible, other variables in the study database predictive of the missing values or influencing the cause of missing data will be included. For the sensitivity analyses addressing missing not at random, if assessment of reasons for missing data suggests that data may be systematically related to unobserved values (eg, inability to attend the next day assessment due to severe AT pain), sensitivity analysis will also be performed to assess the potential impact of including missing not at random data in the analysis. Methods for sensitivity analysis such as marginal delta adjustment, conditional delta adjustment, reference-based controlled imputation, and other pattern-mixture models will be considered. If needed, variables will be transformed to satisfy approximate normality before imputation and then retransformed to the original scale.

### Data Management

Data will be captured primarily via REDCap, a secure online data management system developed for building and managing online surveys and databases, and compliant with HIPAA and 21 Code of Federal Regulations Part 11. Electronic records collected will be retained for a minimum of 6 years after the completion of the study.

### Ethical Considerations

This study was approved by the University of Iowa Biomedical-01 Institutional Review Board (202405285) on September 19, 2024. During the screening process of this study, potentially eligible participants will be given the study information sheet and electronic consent form, with all questions answered and time provided for review. A copy of the electronically signed consent form will be obtained and kept within REDCap to document compliance, and a copy of the informed consent will be emailed to each participant. Participants will be compensated US $50 for completion of in-person consent and baseline measures, US $15 for each remote survey evaluation (up to US $45 total), US $50 for each remote video evaluation (up to US $150 total), and US $50 for focus group participation. All participant data will be deidentified and assigned a study-specific ID. Electronic records will be stored on University of Iowa network drives and will be accessible only to authorized personnel using password-protected computers.

## Results

This research was funded in July 2024. Participant enrollment began in August 2025 and is expected to conclude in 2028. As of January 2026, 15 participants have been enrolled at the UIHC, meeting the recruitment goals.

## Discussion

Exercise and education have been shown to be effective for the management of AT [4,23,24,26,28,60,61], but to date, no studies have been conducted to gauge the minimal effective dosage of exercise and education interventions or to identify prognostic factors for success with noninvasive treatments in patients with AT. This randomized controlled trial will identify a rehabilitation program focused on education and exercise for AT that expands access to care and will identify factors that predict responses to rehabilitation. This will be accomplished by determining the efficacy of a single-visit, PT-initiated rehabilitation program compared to a multivisit, PT-guided rehabilitation program for AT and by identifying early prognostic factors for individuals who experience the greatest improvement in pain and disability by 4 weeks. 

This trial comprises a robust sample of both midportion and insertional AT. A large heterogeneous sample (N=160) will provide adequate power to detect multiple factors that most strongly predict the success of rehabilitation for AT. Including both subtypes of AT and a range of symptom duration (acute vs chronic) will better represent the natural heterogeneity of the population with AT presenting for care and facilitate the ability to detect potential predictors. Maximizing patient heterogeneity in prognostic studies allows models to account for a wider range of characteristics and factors that may influence outcome prediction, enhancing generalizability to diverse real-world populations and clinical settings [62].

## Supplementary material

10.2196/90955 Peer Review Report 1 Peer review report by the US Department of War, Congressionally Directed Medical Research Programs (HT94252410536)

## Abbreviations

AT: Achilles tendinopathy
CRDAMC: Carl R. Darnall Army Medical Center, Fort Hood
HIPAA: Health Insurance Portability and Accountability Act
NRS: numeric rating scale
PACE: Progressive Achilles Loading via Clinician e-Support
PT: physical therapist
REDCap: Research Electronic Data Capture
SPIRIT: Standard Protocol Items: Recommendations for Interventional Trials
UIHC: University of Iowa Hospitals and Clinics
VISA-A: Victorian Institute of Sport Assessment–Achilles
