# Supplementary material for: Progressive Achilles Loading via Clinician e-Support (PACE): Protocol for a Dual-Site Randomized Controlled Trial
Source: JMIR Res Protoc. 2026 Apr 7;15:e90955. doi: 10.2196/90955 (PMC13056041; doi:10.2196/90955)
Supplement: Peer Review Report 1 [file resprot-v15-e90955-s001.pdf]

Department of Defense
U.S. Army Medical Research and Development Command
Congressionally Directed Medical Research Programs
Fiscal Year 2023 Peer Reviewed Orthopaedic Research Program
Clinical Trial Award - Research Level 1
Peer Review Summary Statement

CDMRP Log Number: OR230004
Grants.gov ID Number: GRANT13974073
Review Panel: Clinical Trial - 2
Discussion Period: 11/06/2023-11/07/2023

Project Duration: 48 months
Budget Requested: $2,499,776
Direct Costs: $1,865,357
Indirect Costs: $634,419

Title: Expanding and Accelerating Access to Care for Achilles Tendinopathy Through Self-Management Rehabilitation Strategies
Principal Investigator: Ruth Chimenti
Performing Organization: Iowa, University of
Contracting Organization: Iowa, University of

OVERVIEW

The Principal Investigator (PI) of this application proposes to advance rehabilitation for Achilles tendinopathy (AT) by providing the right care at the right time rather than simply administering more care. The objectives of this noninferiority trial are to identify a rehabilitation program focused on education and exercise for AT that expands access to care for military personnel and to identify factors that predict responses to rehabilitation. The study hypothesis is that a single-visit, physical therapist (PT)-initiated rehabilitation program will be as effective in improving pain and reducing disability as a multivisit, PT-guided rehabilitation program for AT and that within 2 weeks of initiating exercise treatment, early changes in patient-reported outcomes can identify responders. The specific aims are to (1) determine the efficacy of a single-visit, PT-initiated rehabilitation program vs a multivisit, PT-guided rehabilitation program for AT and (2) identify early prognostic factors for individuals who experience the greatest improvement in pain and disability by 4 weeks. A single-blind, 2-arm randomized controlled trial will be performed. The intervention is single-visit, PT-initiated therapy vs multivisit PT-guided therapy, and the projected results are noninferiority of the single-visit therapy.

	Average Score	Standard Deviation
**Overall Evaluation**<br>*Rating Scale: 1.0 (highest merit) to 5.0 (lowest merit)*	1.7<br>**(Excellent)**	0.1
**Criteria**<br>*Rating Scale: 10 (highest merit) to 1 (lowest merit)*	**Average Score**	
**Research Strategy**	9.0	
**Intervention**	8.7	
**Recruitment, Accrual, and Feasibility**	8.3	
**Impact and Military Benefit**	7.1	
**Statistical and Data Analysis Plan**	8.3	
**Ethical Considerations**	9.0	
**Regulatory Strategy and Transition Plan**	9.6	
**Personnel and Communication**	9.3	

SCORED CRITERIA

Research Strategy
Average Score: 9.0

Scientist Reviewer A

Strengths: The scientific rationale for the proposed study based on pathology prevalence, existing treatment strategies, and the benefits of telehealth in this setting is very robust. AT is a common malady affecting both the civilian and military population, although significantly more prevalent in military personnel. While the noninvasive management of this problem is well established, the delivery of the treatment protocol is not. With advances in care delivery via telehealth and virtual platforms, efficient delivery of health care is being optimized both on the patient and practitioner side. This study aims to do exactly this for patients with AT. Evidence from other musculoskeletal pain conditions indicates that a single visit with a PT that includes targeted education and exercise may be as effective as multiple individual physical therapy visits at providing equivalent pain relief, improving function, and restoring quality of life. The study questions, specific aims, hypotheses, and objectives are clear and concise. The experimental design, methods, data collection procedures, and analyses are designed to answer the clinical objective and purpose robustly. The outcome measures to be utilized are appropriate and well supported/validated in previous high level studies. The inclusion/exclusion criteria are well suited to the study and are optimized to the target population. Likewise, the data collection instruments are validated and appropriate to measure outcomes sought by this study. In summary, this is a robustly designed study all around. The applicants have proposed a methodologically sound study.

Weaknesses: Some patients with AT may require treatment for longer than 6 weeks for resolution of symptoms. While this study equips patients with the knowledge and strategies to continue self-guided physical therapy, it does not address the potential need for ongoing individualized therapy programs for those who may benefit from this.

Scientist Reviewer B

Strengths: The use of a noninferiority trial is appropriate for the target population within the application and its hypotheses/objectives, which are to identify a rehabilitation program focused on education and

exercise for AT that expands access to care for military personnel and to identify factors that predict responses to rehabilitation. The hypothesis that a single visit of PT rehabilitation program for AT will be as effective in improving pain and reducing disability as a multivisit, within 2 weeks of initiating exercise treatment on patient reported outcome measures is clear and in support of the clinical objective and purpose for the target population. The study design is a single-blind, 2-arm randomized controlled trial. Individuals with AT will be randomized to 1 of 2 rehabilitation programs (single visit, vs standard of care). The single visit will be conducted via telehealth with asynchronous modules, vs the multivisit (standard of care) will be conducted via 6 one-on-one telehealth visits. The experimental design to include 160 individuals with AT from a medical center, as well as the University of Iowa Medical Center, improves the applicability to the target population. The follow-up time points from baseline of 2 and 4 weeks, as well as 8 weeks, 26 weeks, and 52 weeks improve the capacity for short and long term outcomes to be assessed. Specific Aim 1 is to determine the efficacy of a single visit PT initiated rehabilitation program vs a multivisit PT guided rehabilitation program for AT. The hypothesis is that the single visit will be noninferior for AT to relieve pain, and reduce disability. Primary outcomes for this aim are numeric pain rating scale and the disability with the Victorian Institute of Sports Assessment-Achilles (VISA-A). The outcomes are standard, valid, and reliable instruments and are appropriate for the aims and objectives of the study at 4 weeks. Specific Aim 2 is to identify early prognostic factors for individuals who experience the greatest improvement in pain and disability by 4 weeks. The hypothesis is that individuals with greater immediate (within 2 weeks) improvement in self-efficacy, pain, and disability will obtain the greatest benefits from AT-targeted education and exercise.

Secondary outcomes include ultrasound as a prognostic marker (preintervention), military readiness, and a variety of health domains. These are exploratory and appropriate as prognostic markers (Patient-Reported Outcomes Measurement Information System, Tampa Scale of Kinesiophobia-17, Optimal Screening for Prediction of Referral and Outcome-Yellow Flag tool, Patient Global Rating of Change, Foot and Ankle Ability Measure, General health-based 36-Item Short Form-36). Research Electronic Data Capture (REDCap) will be used to collect secured data. The specific aims and hypotheses are well supported within the background and preliminary data and answer clearly the clinical objective and purpose. The inclusion of education to address kinesiophobia is supported by preliminary data. Further, preliminary data support the inclusion of targeted exercise and education focused on self-management and psychological factors, reduced pain and fear of movement, and increased self-efficacy. In a prior randomized controlled trial, a civilian population was used that experienced either insertional or midportion AT expanding the reach of the intervention beyond previous studies that focused only on one or the other.

Preliminary data support the use of telehealth (in comparison to in-person care) for pain, reduction in disability, global rating of change, and therapeutic alliance across 12 weeks. Interventions included education and exercise for both groups. Identifying prognostic factors for success using a heterogeneous sample (AT variety), as well as a larger sample, would increase external validity and utility for clinicians. Preliminary data support body mass index (BMI), VISA-A, and potentially self-efficacy as a predictor for demographics and/or psychosocial models during an exercise plus education intervention at 12 weeks. The target population includes inclusion criteria (AT pain greater than or equal to 3/10 with tendon-loading exercise), and exclusion criteria (<18 years of age, insufficient level of exercise, recent prior physical therapy for AT, risk of falls, systemic inflammatory disease, or logistical problems) are appropriate to be internally valid and externally applied for generalizability.

Weaknesses: There are no preliminary data to support the use of a single visit; however, background from other musculoskeletal injuries (TKA, RCT, whiplash) supports success of a single visit.

Intervention
Average Score: 8.7

Scientist Reviewer A

Strengths: There is strong evidence to support the proposed intervention (a single visit with a PT that includes targeted education and ongoing access to this material) in the conservative management of other musculoskeletal conditions. The intervention directly meets the need of the patient (early access to care, less logistical burden in obtaining care) and those of the health care system (efficient care delivery, decreased direct and indirect costs). The proposed intervention is feasible for use in its intended environment, and end points are rational. The study design will provide evidence to support the safety and efficacy of the intervention in this noninferiority model. The intervention is delivery of a gold standard PT protocol in a novel mode which has been shown successful in the management of other similar musculoskeletal conditions. While the risks of the intervention are extremely low, the clinical monitoring plan is very appropriate. The study design will implement measures to ensure the consistency of care delivery between groups that exceed expectations.

Weaknesses: The effective delivery of the intervention relies in part on the patient's access to high speed internet and a device capable of viewing the resources. While this is well accounted for in the study design, translation of the results to the target population relies on this as well. Albeit a very small segment of the population, some may not have access to the equipment/environment to benefit from the intervention. The efficacy of the intervention depends in part on the patient's inherent willingness and/or intrinsic level of self-motivation to participate in their rehabilitation. While the study's secondary objective is to identify demographic data points that help categorize early/effective responders to the intervention, these demographics are unlikely to account for a subject's inherent willingness and/or intrinsic level of self-motivation, thus potentially limiting this objective. These are minor concerns.

Scientist Reviewer B

Strengths: The telehealth strategy follows current clinical practices from Fort Cavazos, using Medbridge. The rehabilitation protocols are consistent with current clinical practice. Patients will receive care with links to videos, pdfs of handouts, and review questions optimized for access via mobile phones. The intervention from the PT will be guided by a script for consistency and reduced bias. The comparison between the PT-initiated and PT-guided (standard of care) addresses clinical and operational needs. The PT initiated is a single synchronous visit along with 6 asynchronous visits. The PT-guided is 6 one-on-one 30-minute visits with a PT using telehealth, and visits will occur weekly for the first month and biweekly for the second month. The physical intervention program focusing on loading the AT is standard of care. The education program focusing on promoting self-management skills and movement and reducing pain is standard of care. The interventions are feasible for the intended environment and effectiveness of the intervention. The clinical monitoring plan supports reduced risk as well as validity. Treatment fidelity will be supported by training from Dr Chimenti for internal and external validity. There will be 4 treating PTs.

Weaknesses: No weaknesses were noted.

Regulatory Compliance Specialist Reviewer

Strengths: Measures proposed for use in the study are well defined. In particular, for the primary end points, the applicant explains why each of the patient reported outcomes was selected (Numeric Rating Scale and VISA-A); standard deviations and minimally important differences are defined. To maintain blinding, baseline measures will be captured in-person during the consent visit and prior to randomization. To minimize any influence of the treatment provider, performance-based outcomes (pain with activity and physical function) for both groups will be captured remotely by a research assistant blinded to treatment group at 4, 8, 12, 26, and 52 weeks. Access and availability of the intervention are

Procurement Sensitive Information
Do not copy or distribute

not a risk to the study, as the intervention is physical therapy techniques not dependent on specific drug, biologic, or experimental device products (some standard physical therapy assessment tools are utilized).

The proposed trial addresses the clinical and/or operational need(s) in focus area 2b: retention strategy/return to duty, and also focus area 3a: translation of early findings: soft tissue trauma. The proposed intervention is feasible for use in its intended environment. The study design appears likely appropriate to provide evidence to support the safety, efficacy, and/or effectiveness of the intervention, especially for the primary end point. Both rehabilitation programs are consistent with current clinical practice guidelines. The study interventions do not introduce any significant risk to subjects (beyond that expected from standard of care); therefore, preclinical and clinical evidence is not needed to support use of the proposed interventions in this study. The clinical monitoring plan is appropriate to address the level of risk of the clinical trial. Research procedures are clearly delineated from routine clinical procedures.

Weaknesses: There are too many patient-reported outcomes used for secondary end points, with significant overlap/redundancy between these measures. Especially as study subjects are asked to complete these at multiple visits, this is unduly burdensome to subjects, which is a risk factor for retention and compliance. Also, respondent fatigue can adversely impact the quality of data collected. Given the goal of the study, subjects cannot be blinded to their treatment assignment. While unavoidable, this is a potential source of bias for the patient reported outcomes.

Recruitment, Accrual, and Feasibility
Average Score: 8.3

Scientist Reviewer A

Strengths: The application addresses the availability of human subjects for the clinical trial and the prospect of their participation in exemplary detail. They provide specific data from the 2 enrollment sites based off of recent studies of the same population as well as current practice volumes. They demonstrate that delivery of care via telehealth has boosted enrollment in prior studies, arguing that the delivery mode will improve recruitment and participation with low attrition. The proposed study population simulates the targeted patient population that might benefit from the proposed intervention, drawing from both military and civilian populations but with inclusion criteria designed to only enroll the segment of the civilian population that overlaps with the functional levels of the military subjects. The recruitment, informed consent, screening, and retention processes for subjects are appropriate for the proposed clinical trial. The application specifically identifies low enrollment and possible attrition as potential but unlikely problems. They present specific, detailed, and reasonable mitigation plans to resolve them. The study team has experience with mitigating both of these issues successfully in previous clinical trials. The strategy for the inclusion of women and minorities is well detailed and appropriate to the objectives of the study. Likewise, the distribution of the proposed enrollment on the basis of sex/gender, race, and/or ethnicity is very well detailed and appropriate for the proposed research.

Weaknesses: One proposed strategy to mitigate low enrollment is to modify inclusion criteria to lower the minimum pain threshold for inclusion. It is unclear if including patients with a very low pain score will dilute the measured effect of the intervention.

Scientist Reviewer B

Strengths: The use of telehealth will aid in recruitment and retention. However, there is a single-in-person visit for screening and enrollment. Groups will be randomly allocated in a stratified manner based upon AT type and symptom duration for 4 strata per site. The study will purchase tablets to be loaned out to participants for the first 8 weeks of participation. Modification of exclusion criteria is proposed as a

strategy for unexpected recruitment issues. The proposal does demonstrate access to the study population. The screening process and informed consent in person visit are appropriate. The reimbursement is appropriate and should support recruitment and retention. The inclusion of women and minorities is addressed and is representative of the active-duty force supporting the generalizability of the findings.

Weaknesses: The proposal addresses the availability of human subjects within the statistics section. With a history of enrolling 41 subjects per year at Iowa, they anticipate being able to do this at both sites. This may be too optimistic.

Consumer Reviewer

Strengths: Between the 2 proposed centers there should be an ample pool to meet the recruitment requirements. Ft Cavazos is a large military base with both combat arms and combat support units as well as a transient training base. With the number of injuries of the military as compared to the civilian population they should be able to reach the needs of the study. The University of Iowa has a large base of athletes and veteran students that potentially encounter AT, so there should be no recruiting issues. Because age is not an exclusion factor (veteran populations have higher rates than the civilian population) the pool simulates the military and veteran populations well. This proposal seems to have done an in-depth study on both population groups at the Carl R. Darnall Army Medical Center (CRDAMC) and University of Iowa Hospitals and Clinics (UIHC) sites and appears to cover recruiting requirements for FY23 PRORP CTA proposals. This study appears to have a comprehensive plan to include women and minorities. They address these areas of women and minorities, and it appears to be appropriate.

Weaknesses: While this study has a good plan in place to handle many of the pitfalls of a trial like this their knowledge base of how frontline care and battlefield treatments are handled gives them A disadvantage in their goals' achievement.

Impact and Military Benefit
Average Score: 7.1

Scientist Reviewer A

Strengths: The proposed clinical trial directly fills a gap in orthopaedic research and care and is specific to the FY23 PRORP CTA focus area, retention strategies. Specifically, the proposed trial evaluates an intervention that can be applied in a battlefield setting within 30 days of injury to the standard of care, which can be applied along the continuum of care to enable return to duty within 1 year of injury (focus area). AT is a relatively high prevalence musculoskeletal problem in the military population. Early and effective delivery of care to this group will not only improve return to duty, but also provide significantly positive impacts on the health care system, freeing up providers to see and treat other injured personnel. The short and long term economic benefits of the proposed intervention could be significant. Modeling similar intervention strategies for other musculoskeletal injuries could be a major benefit of this study. The impact it may have will be significant for the target population as well as the health care system. The sample population represents the targeted patient population very well (specific effort to include only civilians with functional capacity on par with the military population).

Weaknesses: There may be a subset of subjects within the target population that is low self-motivated. It is unclear if and how this study will account for this.

Scientist Reviewer B

Strengths: AT is common in military personnel, and impact to the target population within the focus area would have great impact. The potential impact of a single visit of physical therapy focusing on education and exercise would support the focus area of retention strategies for optimization and/or rehabilitation strategies that can facilitate retention on duty or avoid reinjury for common combat-related musculoskeletal injuries. The use of telehealth and asynchronous follow-up modules would allow for greater flexibility and accelerated access to care for the target population to complete therapy and restore function. Further, a single visit has potential to improve efficiency and reduce cost. The identification of early prognostic factors will also support clinical decision making. The sample population, including military, along with the inclusion criteria well match the target population.

Weaknesses: No weaknesses were noted.

Consumer Reviewer

Strengths: This clinical trial directly addresses the FY23 PRORP focus area: retention strategies: optimization and/or validation of battlefield-feasible diagnostic capabilities, decision support tools, interventions, and/or rehabilitation strategies that can facilitate retention on duty or avoid reinjury for common combat-related musculoskeletal injuries. An increase in knowledge about the diagnosis care and multiple paths to recovery will impact a soldier's return to duty. This impact will be limited at best under battlefield conditions yet may prove partially impactful once removed from frontline conditions. This proposal could impact the return to duty time frame compared to the current treatment plans. Better tools and training to diagnose and provide early treatment are always a best case scenario for the injured personnel. If this proposal's new methods may support better healing, this would impact the injured population. The concept of providing the right care at the right time is always a positive for all. This proposal has the potential to lower the amount of time being treated and deliver care in a faster way due to remote (telehealth) sessions instead of having to schedule one on one time with PT or medical personnel. Also, the improved diagnostic procedures and faster entry into a PT care program could impact the military and civilian populations. By delivering timely care instead of waiting for scheduling of specialty clinics the impact could be significant. It makes sense that treating quickly could reduce the injury faster and better. This study appears to have access to both civilian care at the University of Iowa and the Darnall Army Medical Center, Fort Cavazos which should allow for a good mix in types of patients and injuries.

Weaknesses: This study believes that the military will be able to use telehealth to better diagnose and then treat all the way to the battlefield. This will actually lower the unit readiness and safety of the injured. Any injury that causes a loss of movement and increased pain will slow warfighters down thus creating a dangerous scenario for themselves and those around them. Keeping an injured person with an injury such as AT is life threatening at the least. There is a reason that units remove injured back to aid stations then higher medical care. This allows a unit to continue their mission without having to slow down or care for warfighters that cannot shoot, move, and communicate at their best level. The need to get back to one's unit is very strong, and a shortened unchecked recovery plan could allow the injured to return to duty prior to a recovery point well enough to keep themselves safe and those around them out of potential issues. The battlefield is the ultimate testing ground. The problem is that lives are lost for a failed test not just reinjuries. This study's impact will be limited unless measures are put in place to ensure full recovery prior to back to duty status is achieved. Less than this will cost lives and hurt mission completions.

Statistical and Data Analysis Plan
Average Score: 8.3

Scientist Reviewer A

Strengths: The statistical model and data analysis plan are appropriate for the planned study. The sample size projection based on the power analysis appropriately, if not over, accounts for expected attrition and is based on primary outcomes. The statistical analysis plan accounts for the possibility of limited clinical enrollment/attrition (beyond expected attrition) with a power analysis using double the expected attrition rate to ensure sufficient sample size at final follow-up. Plans for valid analysis of group differences on the basis of sex/gender, race, and/or ethnicity are described and appropriate for the proposed research.

Weaknesses: A sensitivity analysis on AT type (insertional vs midportion) and AT duration (acute vs chronic) will be assessed to inform potential differences by location of pain within the Achilles tendon although it is unclear if the study is adequately powered to detect meaningful differences among these subpopulations. This is a minor concern as this will not affect validity of main outcome measurements or their generalization to the intended population.

Scientist Reviewer B

Strengths: The statistical model and data analysis plan include summary statistics for the 2 groups. An intention to treat analysis is appropriate, and a noninferiority method is ideal for the objectives of the proposal. Linear regression models to examine predictors of improvement in pain and disability are appropriate and will use an Akaike information criterion for the predictor variables. Power analyses are based upon minimally clinically important differences, dropout, an alpha level of 0.05, and a power of 80%. Together, these summed to have a sample size of 160 to achieve Aim 1B. This will account for anticipated attrition. Missing data will be compensated for using a multiple imputation of missing values. The plan compensates with increased enrollment to assess subpopulations, by design. There are no plans for group differences based upon sex/gender, race, and/or ethnicity as is appropriate for this study design.

Weaknesses: It is unclear why 2-sample *t* tests, or nonparametric tests, are proposed to assess heterogeneity. Secondary models to assess predictors for AT-type and -duration, and sex are not described. Minimal detectable change, and/or minimally clinically important difference values, is not established and/or implemented for both groups to assess intervention effectiveness for both groups. The use of BMI, VISA-A, and potentially self-efficacy as a predictor for demographics and/or psychosocial models during an exercise plus education intervention at 12 weeks is potentially limited due to inadequately powered sample size issues.

Biostatistician Reviewer

Strengths: The statistical analysis plan is appropriate overall given the study design with clearly defined primary and secondary end points with respect to assessment time points. For Aim 1 linear mixed models are clearly specified with potential fixed and random effects. Model building strategy for Aim 2 is clearly defined. Secondary analysis and the way to handling missing values are appropriate too. Power analysis is adequate with all necessary parameters clearly defined. Particularly the noninferiority margins are properly defined with support from previous literature.

Weaknesses: For Aim 2, it is not clear how nonlinearity functional can be detected in linear regression models. Also, it is not clear how the effect size f^2 is defined for power analysis.

Ethical Considerations
Average Score: 9.0

Scientist Reviewer A

Strengths: Existing evidence demonstrates the study interventions are consistent with sound research design and, when appropriate, that these procedures are already in use for treatment of AT and other musculoskeletal conditions. The population selected to participate in the trial stands to benefit significantly from the knowledge gained. The proposed clinical trial might affect the daily lives of the individual human subjects participating in the study significantly by providing earlier access to care and less logistical burden in receiving it. The process for seeking informed consent is appropriate. The level of risk involved with the proposed intervention is extremely low. The safety monitoring and reporting plan is well described and exceeds the level of risk. Privacy and confidentiality issues are appropriately considered.

Weaknesses: No weaknesses were noted.

Scientist Reviewer B

Strengths: The study procedures are consistent with a sound research design, and are already standard of care for treatment purposes. The proposal is to compare noninferiority of existing care models. The population selected to participate does stand to benefit from the knowledge gained, as AT may come back for any of them in the future. Further, they will be honored for their time commitment. The daily lives of participants will be minimally impacted, and improved in that they will be monitored more closely than if they were under the usual care model. The process for seeking informed consent, in person, is appropriate, and there are no vulnerable populations included. The level of risk to subjects is minimized; standard of care, and safety monitoring by Dr Hall is appropriate. The risk management and emergency response is appropriate. There are minor risks associated with treatment and screening; however, these are not greater than normal activities should an individual meet inclusion/exclusion criteria. The use of REDCap for data collection is appropriate for confidentiality. There are no international sites included.

Weaknesses: No weaknesses were noted.

Bioethicist Reviewer

Strengths: The proposed prospective randomized controlled trial aims to identify factors predictive of improved patient outcomes with AT while comparing a single-visit, PT-initiated rehabilitation program to a multivisit, PT-guided rehabilitation program with the use of telehealth for military service members. The scope of the problem (lack of prognostic factors to determine which patients will benefit from rehabilitation and which should be directed toward more invasive procedures) is compelling, and the use of telehealth is well justified considering supporting evidence of accessibility and efficacy, especially in the wake of Covid-19.

Weaknesses: Several ethical issues are notable in participant selection, assignment, and privacy/confidentiality of this study. The research team proposes to recruit service members as well as civilians who exercise a comparable amount to service members, veterans, and reservists. However, it is not clear whether these 2 groups can be compared effectively without threatening internal validity. The logistical and resource burdens seem substantially higher for participants assigned to the multivisit program than for the single visit program. The applicants do not comment on the effects that these added burdens might have on the study results. It is also unclear how providers/clinicians as well as participants would remain blinded as the interventions have obvious differences. For example, patients would need to schedule multiple appointments (six 30-minute appointments) vs one. To the extent the single blinding is a critical design feature of the trial, the investigators may have difficulty maintaining it throughout the study and could bias patient reported outcomes/results. Lastly, for those enrolled participants who are on

active duty or in the field, there is unlikely to be an appropriately quiet/private place to conduct telehealth visits that protects their confidentiality.

Regulatory Strategy and Transition Plan
Average Score: 9.6

Scientist Reviewer A

Strengths: The rehabilitation interventions used in this clinical trial do not require regulation by the FDA or other regulatory body, because the trial does not evaluate a test product. Because this is a low-risk intervention intended to gain generalizable knowledge without product commercialization aims, it qualifies for exemption from IND/IDE requirements. As a potential knowledge product, the pathway to provide continuity of development, including proposed development or modification of clinical practice guidelines and recommendations, provider training materials, patient brochures and other clinical support tools, scientific journal publications, models, simulations, and applications, is described and achievable. The schedule and milestones for bringing the intervention to incorporation into clinical practice are reasonable. The study group intends the clinical resources developed from this clinical trial (handouts and videos, etc) to be publicly available allowing free use with attribution. They do not anticipate the development of new intellectual or material property. This approach will maximize accessibility and downstream benefits. This team is well experienced in leveraging resources to maximize a transitional plan. This is well supported in their previous work and in this application.

Weaknesses: No weaknesses were noted.

Scientist Reviewer B

Strengths: The regulatory strategy is a knowledge product, and is not FDA regulated. The application states that the intervention is low-risk and qualifies for exemption from IND/IDE requirements. The transition plan is to publish and present to open access platforms, propose a follow-up clinical trial in a battlefield setting, and then perform knowledge translation within the American Physical Therapy Association clinical practice guideline. These are appropriate for knowledge products and are achievable as the PI is currently on the clinical practice guideline group. Funding strategies include DOD PRORP CTA, VA, NIH, foundations, and industry partners for e-platform distribution. This group appears to be successful in funding, and the mechanisms described would allow for the intervention to go to the next level of development. Intellectual property (IP) will be publicly available allowing for free use and translation. There is a plan for IP.

Weaknesses: No weaknesses were noted.

Regulatory Compliance Specialist Reviewer

Strengths: The regulatory strategy and transition plan are appropriate for the proposed study. There are appropriate plans for IRB review/oversight, including designation for the central IRB of record. The application correctly states that the research does not involve any investigational device, or a drug or biological product as defined in US law; therefore, IDE and IND submission is neither required, nor is either of them appropriate. Proposed collaborations and other resources appear sufficient to support the proposed knowledge products. Plans for good clinical practice compliance (including IRB oversight) are appropriate. Good manufacturing practice and good laboratory practice are not applicable as the applicant is not manufacturing intervention products, and no animal trials are included. Proposed development of knowledge products is achievable. The schedule and milestones are realistic and achievable. The transition plan is appropriate. The applicant correctly states that IP is not applicable to development of

knowledge products through this trial. There are no applicable concerns regarding conflicts of interest. Risk analysis for cost, schedule, manufacturability, and sustainability is generally appropriate. The applicants acknowledge that risks to successful translation include limited clinician buy-in and patient adherence; this awareness is an important first step to effectively mitigating this risk.

Weaknesses: The trial plans for a data safety and monitoring board; this does not seem warranted, since all subjects will receive standard of care.

Personnel and Communication
Average Score: 9.3

Scientist Reviewer A

The PI, Ruth Chimenti, received a DPT in 2009 from Washington University, St Louis and a PhD in health practice research in 2014 from the University of Rochester and is an assistant professor in the Department of Physical Therapy & Rehabilitation Science at the University of Iowa, Iowa City, Iowa.

Coinvestigator, Jason Wilken, received a PhD in physical rehabilitation science in 2006 from the University of Iowa and is an associate professor of physical therapy at the University of Iowa.

Coinvestigator, Mederic Hall, received an MD in 2006 from the University of Illinois College of Medicine, Chicago and is a professor of orthopaedics and rehabilitation at the University of Iowa.

Coinvestigator, Patrick Ten Eyck, received a PhD in biostatistics in 2015 from the University of Iowa and is assistant director for biostatistics and research at the University of Iowa.

Co-PI, Mathew Frazier, received a DPT in 2009 from Creighton University and is a PT at the Carl R. Darnall Army Medical Center, Fort Cavazos, Texas.

Strengths: The composition of the study team is well balanced and appropriate for the proposed study. The study personnel and organization are very clearly laid out in the proposal. The study team's background and expertise are very appropriate to accomplish the proposed work. They have experience running large clinical trials in both the military and civilian populations and significant experience with the pathology being studied and its treatment. They demonstrate a positive track record with recruitment, retention, safety monitoring, and statistical analysis. The levels of effort of the study team members are appropriate for successful conduct of the proposed trial. The logistical aspects of the proposed clinical trial (communication plan, data transfer and management, standardization of procedures) are very clearly explained. This is a relatively small study group with study conducted at only 2 sites, so the logistical aspects of the study are quite simple. The lead site responsibilities and human research protections regulatory coordination are well defined and clearly planned for.

Weaknesses: The study PI has experience participating in similar large clinical trials but with limited experience as a study PI. This is a minor concern given her otherwise robust resume.

Scientist Reviewer B

Strengths: Dr Ruth Chimenti, at the University of Iowa, physical therapy and rehabilitation science (assistant professor) is an expert within the field of AT and is experienced with multisite clinical trials. Dr Mathew Frazier, at the Carl R. Darnall Army Medical Center has experience with multisite trials and evaluation of physical performance of the lower limbs within the military population. Dr Patrick Ten Eyck, at the University of Iowa is a biostatistician. Dr Jason Wilken, at the University of Iowa has

experience with multisite trials and evaluation of physical performance of the lower limbs within the military population. Dr Mederic Hall, at the University of Iowa is a physician, who has collaborated with Dr Chimenti in the past and is a safety officer with expertise in AT. The composition of the team is appropriate for the work proposed. The group appears to have worked together in the past, and been successful. The levels of effort and milestones are appropriate for the proposed work. The site responsibilities across the 2 locations are well defined, and the human subjects protections are accounted for at both. Letters from the Coinvestigators are appropriate and supportive of accomplishing the project. The organizational chart, communication logistics, and timetable for the 2 sites are provided. The levels of effort of the study team members are appropriate for successful conduct of the proposed trial.

Weaknesses: This will be the first time the PI will lead a large clinical trial, a minor concern given prior experience.

UNSCORED CRITERIA

Environment

Scientist Reviewer A

The scientific environment, clinical setting, and accessibility of institutional resources robustly support the clinical trial at both participating centers. There is very strong evidence for appropriate institutional commitment from each participating institution.

Scientist Reviewer B

The University of Iowa is well suited to perform the proposed work. The UIHC is a large hospital that supports the sample size anticipated and the target population. The Department of Physical Therapy at the University of Iowa has ultrasound imaging allowing the PI to collect baseline metrics. Institution resources are excellent. The CRDAMC is also well suited to perform the proposed work, and the environment is outstanding. The letter from the professor and chair of the University of Iowa Physical Therapy Department is supportive of the project.

Budget

Scientist Reviewer A

The budget is appropriate for the proposed clinical trial.

Scientist Reviewer B

The budget is appropriate for the proposed work and is less than the allowable costs.

Application Presentation

Scientist Reviewer A

This is a very well-written, well-designed, well-supported application.

Scientist Reviewer B

The application writing, clarity, and presentation of the proposed work supported the evaluation of the proposal.
